# Climate change can be seen through a disaster medicine lens

**DOI:** 10.5694/mja2.51730

**Published:** 2022-10-09

**Authors:** George Braitberg

**Affiliations:** ^1^ University of Melbourne Melbourne VIC; ^2^ Austin Health Melbourne VIC

**Keywords:** Disasters, Climate change, Evidence‐based medicine, Disaster medicine

Climate change is real and the scale of disasters that it causes will increase

The World Health Organization defines disaster as “any occurrence that causes damage, ecological disruption, loss of human life or deterioration of health and health services on a scale sufficient to warrant an extraordinary response from outside the affected community or area”.^1^The impact of a disaster is determined by three factors: the hazard itself (an extreme event that can be natural or man‐made), the vulnerability of the affected people, and the capacity or measures that can be taken to reduce or cope with the potential negative consequences. A disaster occurs when the hazard exposes the vulnerability of individuals and communities in such a way that their lives are directly threatened, or sufficient harm has been done to their community’s economic and social structure to undermine their ability to survive. Harm can be defined as loss of life, injury, disease, and negative effects on human physical, mental and social wellbeing, together with damage to property, destruction of assets, loss of services, social and economic disruption, and environmental degradation. Vulnerability refers to the person’s or population’s susceptibility to be harmed and their capacity to anticipate, cope with, and recover from the impact of a hazardous event.^2^ Importantly, vulnerability is not uniform and is determined by access to economic and material resources, human or personal resources (such as education), family and social resources (such as networks of reciprocity), and political resources (such as power and autonomy). The more general the disaster, the more likely disadvantaged communities will suffer.^3^, ^4^, ^5^, ^6^ Mitigating factors that reduce the potential impact include prevention measures, community preparedness, and resilience. Once a disaster occurs, its impact can be determined by applying the following formula:

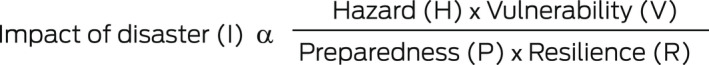

## Impact (I)

The frequency of international disasters has grown from fewer than ten events per year in the mid‐20th century to several hundred per year.^7^ The cause for this growth is multifactorial. The contribution of human behaviour, urbanisation, overcrowding, and the increasing interconnectedness of the world appears to be responsible for the spread of epidemics such as human immunodeficiency virus (HIV) infection, severe acute respiratory syndrome (SARS), avian influenza, Middle East respiratory syndrome (MERS), and coronavirus disease 2019 (COVID‐19).^8^ Higher temperatures increase the frequency of vector‐borne infectious diseases, such as Ross River virus and dengue fever, due to changes in vector biology — shorter larval period and accelerated viral replication time.^9^ Atmospheric changes are linked with extreme weather events. Higher ocean temperatures with rising sea levels, together with more powerful storms, account for increases in coastal flooding, and elevated temperatures cause droughts, degrade agriculture and result in more and larger bushfires.^7^, ^10^, ^11^, ^12^ In Australia, land areas have warmed by around 1.4°C since 1910.^13^ Flood clusters, such as those that have affected the town of Lismore, New South Wales, throughout February and March 2022 are caused by the so‐called El Niño Southern Oscillation (ENSO), when extended periods of dry weather of El Niño render the soil less capable of absorbing the heavy rains during the La Niña event. If greenhouse gas emissions continue to rise at the current rate, extreme El Niño and La Niña events are predicted to double in frequency by the end of the 21st century.^14^, ^15^

## Hazard (H)

There is currently no declaration of a climate emergency at the federal level in Australia, although many jurisdictions have taken this step.^16^ The inability to clearly define the hazard hampers efforts to deal with it. Seen through a disaster medicine lens, we are approaching a “perfect storm”; we have a complex hazard that is affecting a fatigued community with lowered resilience, without agreement on a comprehensive set of measures that can be taken to mitigate the impact. Even among the signatories of the Paris Agreement there is no consensus on how to limit global warming to below 1.5°C.^17^ Simply agreeing on an outcome does not prevent a disaster from occurring.

## Preparedness (P)

To reduce the impact of climate change, we need systems in place that address the core problem and assist in managing the related disaster events. We should stop blaming “nature” or construe them as “acts of god” so that we can prepare accountable, proactive preventive approaches to reduce their impact.^18^ Attributing disasters to nature provides a convenient rationalisation for individuals whose role is to develop policy and the strategic and instrumental measures needed to reduce the risk, hazard and vulnerability impact and improve community resilience.^19^ Hurricane Katrina is an example of the “failure of man” to address the destruction of the city’s natural flood protection through poor project planning, flawed project design, and misplaced priorities that disproportionally affected the most vulnerable.^20^

## Vulnerability (V)

Vulnerability is a product of social and political processes arising from poor governance.^19^ It increases when there is a major disaster or when there are recurrent small or medium scale events affecting the same community. In consideration of the latter, there is emerging research of consequences of the various restrictions put in place in each Australian jurisdiction under public health emergency powers in response to the COVID‐19 pandemic.^21^

## Resilience (R)

Since December 2019, Australia has faced continuous challenges to community resilience. The 2019–2020 bushfires burned over 17 million hectares across the country, which was the largest area in a single recorded fire season for eastern Australia.^22^ Tragically, 34 people died, and the losses of $1.9 billion in insurance claims exceeded the Black Saturday fires of 2009.^23^ To 12 September 2022, Australia has reported 10 112 229 confirmed cases of COVID‐19, with 14 421 deaths.^24^ It would be fair to say that our community and health care worker resilience to withstand further disasters is challenged; a consideration that is important when we consider our capacity to respond to, and recover from, climate change‐driven disasters over the next decades.

As natural disasters are expected to rise, so too are man‐made disasters, such as terror events. Since 2000, there has been a sustained rise in the national security‐oriented literature linking climate change and terror events. Climate change is a threat multiplier leading to community displacement, increasing vulnerability, and facilitating the emergence of extremist views in communities with fragile natural resources, providing terrorists with more opportunity to cause greater damage.^25^

Climate change is real, and the scale of disasters will increase. Our efforts to prepare and respond must be evidence‐based. We must focus on community support and recovery, establishing targeted programs for vulnerable population which provide sustainable financial security and social connectedness.

## Open access

Open access publishing facilitated by The University of Melbourne, as part of the Wiley – The University of Melbourne agreement via the Council of Australian University Librarians.

## Competing interests

No relevant disclosures.

## Provenance

Commissioned; externally peer reviewed.

## References

[1] World Health Organization . Community emergency preparedness: a manual for managers and policy‐makers. WHO, 1999. http://whqlibdoc.who.int/publications/9241545194.pdf (viewed Sept 2022).

[2] Du Y , Ding Y , Li Z , Cao G . The role of hazard vulnerability assessments in disaster preparedness and prevention in China. Mil Med Res 2015; 2: 27. 26605045 10.1186/s40779-015-0059-9 PMC4657270

[3] Morrow BH . Identifying and mapping community vulnerability. Disasters 1999; 23: 1‐18. 10204285 10.1111/1467-7717.00102

[4] Maltz M. Caught in the eye of the storm: the disproportionate impact of natural disasters on the elderly population in the United States. Elder Law Journal 2019; 27: 157‐186.

[5] Elliot JR , Pais J . Race, class, and Hurricane Katrina: social differences in human responses to disaster. Social Science Research 2006; 35: 295‐321.

[6] Raju E , Dutta A , Ayeb‐Karlsson S . COVID‐19 in India: Who are we leaving behind? Prog Disaster Sci 2021; 10: 100163. 34095809 10.1016/j.pdisas.2021.100163 PMC7989097

[7] Harrison CG , Williams PR . A systems approach to natural disaster resilience. Simul Model Pract Theory 2016; 65: 11‐31. 32288694 10.1016/j.simpat.2016.02.008 PMC7118455

[8] Morens DM , Fauci AS . Emerging pandemic diseases: how we got to COVID‐19. Cell 2020; 182: 1077‐1092. Erratum in: *Cell* 2020; 183: 837. 33125895 10.1016/j.cell.2020.10.022 PMC7598893

[9] Naish S , Dale P , Mackenzie JS , et al. Climate change and dengue: a critical and systematic review of quantitative modelling approaches. BMC Infect Dis 2014; 14: 167. 24669859 10.1186/1471-2334-14-167 PMC3986908

[10] Sobel AH , Camargo SJ , Hall TM , et al. Human influence on tropical cyclone intensity. Science 2016; 353: 242‐246. 27418502 10.1126/science.aaf6574

[11] Trenberth K , Dai A , van der Schrier G , et al. Global warming and changes in drought. Nature Clim Change 2014; 4: 17‐22.

[12] Westerling AL , Bryant BP . Climate change and wildfire in California. Climatic Change 2008; 87: 231‐249.

[13] Intergovernmental Panel on Climate Change . IPCC regional fact sheet Australasia. https://www.ipcc.ch/report/ar6/wg1/downloads/factsheets/IPCC_AR6_WGI_Regional_Fact_Sheet_Australasia.pdf (viewed July 2022).

[14] Cook M. Why can floods like those in the Northern Rivers come in clusters? The Conversation 2022; 31 Mar. https://theconversation.com/why‐can‐floods‐like‐those‐in‐the‐northern‐rivers‐come‐in‐clusters‐180250 (viewed Sept 2022).

[15] National Oceanic and Atmospheric Research . How will climate change El Niño and La Niña? Silver Spring, MD: NOAA, 2020. https://research.noaa.gov/article/ArtMID/587/ArticleID/2685/New‐research‐volume‐explores‐future‐of‐ENSO‐under‐influence‐of‐climate‐change (viewed May 2022).

[16] Nissen S , Cretney R . Retrofitting an emergency approach to the climate crisis: A study of two climate emergency declarations in Aotearoa New Zealand. Environment and Planning C: Politics and Space 2022; 40: 340‐356.

[17] United Nations . Climate Action: the Paris Agreement. https://www.un.org/en/climatechange/paris‐agreement (viewed Apr 2022).

[18] Raju E , Boyd E , Otto F . Stop blaming the climate for disasters. Commun Earth Environ 2022; 3: 1.

[19] Lavell A , Oppenheimer M , Diop C , et al. Climate change: new dimensions in disaster risk, exposure, vulnerability, and resilience. In: Field C , Barros V , Stocker TF , Dahe Q ; editors. Managing the risks of extreme events and disasters to advance climate change adaptation: special report of the Intergovernmental Panel on Climate Change Cambridge: Cambridge University Press, 2012; pp. 25‐64.

[20] Shader E. Preventing another “unnatural disaster” ten years after Hurricane Katrina. American Rivers, 2015. https://www.americanrivers.org/2015/08/preventing‐another‐unnatural‐disaster‐ten‐years‐after‐hurricane‐katrina/ (viewed Apr 2022).

[21] Gashaw T , Hagos B , Sisay M . Expected impacts of COVID‐19: considering resource‐limited countries and vulnerable population. Front Public Health 2021; 9: 614789. 34026704 10.3389/fpubh.2021.614789 PMC8131657

[22] Australian Government, Bureau of Meteorology . Annual Climate Statement 2019. http://www.bom.gov.au/climate/current/annual/aus/2019/ (viewed Apr 2022).

[23] Center for Disaster Philanthropy . 2019–2020 Australian bushfires [website]. CDP, 2020. https://disasterphilanthropy.org/disasters/2019‐australian‐wildfires/ (viewed Apr 2020).

[24] World Health Organization . Health emergency dashboard: COVID‐19 — Australia situation [website]. https://covid19.who.int/region/wpro/country/au (viewed Apr 2020).

[25] Asaka JO . Climate change–terrorism nexus? A preliminary review/analysis of the literature. Perspectives on Terrorism 2021; 15: 81‐92.

